# Structural basis for early-onset neurological disorders caused by mutations in human selenocysteine synthase

**DOI:** 10.1038/srep32563

**Published:** 2016-08-31

**Authors:** Anupama K. Puppala, Rachel L. French, Doreen Matthies, Ulrich Baxa, Sriram Subramaniam, Miljan Simonović

**Affiliations:** 1 Department of Biochemistry an Molecular Genetics, University of Illinois at Chicago, Chicago, Illinois 60607, USA; 2 Laboratory of Cell Biology, Center for Cancer Research, National Cancer Institute, National Institutes of Health, Bethesda, MD 20892, USA.

## Abstract

Selenocysteine synthase (SepSecS) catalyzes the terminal reaction of selenocysteine, and is vital for human selenoproteome integrity. Autosomal recessive inheritance of mutations in SepSecS–Ala239Thr, Thr325Ser, Tyr334Cys and Tyr429*–induced severe, early-onset, neurological disorders in distinct human populations. Although harboring different mutant alleles, patients presented remarkably similar phenotypes typified by cerebellar and cerebral atrophy, seizures, irritability, ataxia, and extreme spasticity. However, it has remained unclear how these genetic alterations affected the structure of SepSecS and subsequently elicited the development of a neurological pathology. Herein, our biophysical and structural characterization demonstrates that, with the exception of Tyr429*, pathogenic mutations decrease protein stability and trigger protein misfolding. We propose that the reduced stability and increased propensity towards misfolding are the main causes for the loss of SepSecS activity in afflicted patients, and that these factors contribute to disease progression. We also suggest that misfolding of enzymes regulating protein synthesis should be considered in the diagnosis and study of childhood neurological disorders.

*O*-phosphoseryl-tRNA^Sec^:selenocysteinyl-tRNA^Sec^ synthase (SepSecS; *SEPSECS*) is an ubiquitously expressed enzyme that catalyzes the terminal reaction of selenocysteine synthesis. SepSecS converts the phosphoseryl (Sep) group into the selenocysteinyl (Sec) moiety in a mechanism that requires selenocysteine tRNA (tRNA^Sec^) and a pyridoxal-5-phosphate (PLP) cofactor. The product of SepSecS catalysis, Sec-tRNA^Sec^, is an obligate substrate for selenoprotein synthesis, thus suggesting that the catalytic activity of SepSecS is indispensable for the human selenoproteome integrity.

Recently, two research groups reported that four distinct mutations in human *SEPSECS* caused congenital cerebellar atrophy termed pontocerebellar hypoplasia type 2D (PCH2D). Pontocerebellar hypoplasia (PCH) is a group of autosomal recessive disorders affecting different cerebral structures, particularly the brainstem and cerebellum. Most PCH types result from mutations in genes important for tRNA splicing and aminoacylation and RNA transport–*EXOSC3* in PCH1^1^, *TSEN* and *SEPSECS* in PCH2 and PCH4^2^,^3^,^4^, *RARS2* in PCH6^5^,^6^,^7^, and *CLP1* in PCH10^8^,^9^. In the first report, genome-wide sequencing revealed that patients were either compound heterozygous for Ala239Thr (A239T) and Tyr334Cys (Y334C) or homozygous for Y334C in *SEPSECS*^3^. Although unaffected at birth, patients rapidly presented profound mental retardation, progressive microcephaly, severe spasticity, and seizures and typically survived to only 10–12 years of age^10^. Magnetic resonance imaging found evidence of progressive cerebellar atrophy followed by cerebral atrophy of both white and gray matter. In the second study, whole-genome sequencing revealed children suffering from progressive encephalopathy were compound heterozygous for missense Thr325Ser (T325S) and nonsense Tyr429* (Y429*) mutations in *SEPSECS*^4^. These patients similarly suffered from progressive cerebellar and cerebral atrophy, neonatal irritability, and debilitating spasticity. Neuropathological analysis revealed severe atrophy of the brainstem and cerebellar cortex with loss of both white and gray matter. This subset of patients also exhibited a slight reduction in selenoprotein levels, suggesting that SepSecS catalysis was impaired.

Given that *SEPSECS* is responsible for the formation of only 25 human proteins, its involvement in the development and progression of PCH2D is perplexing. The human selenoproteome is pivotal for the maintenance of the cellular redox potential (e.g. thioredoxin reductases), regulation of the overall metabolic rate (e.g. iodothyronine deiodinases), removal of reactive oxygen species and prevention of oxidative damage (e.g. glutathione peroxidases; GPx, and methionine sulfoxide reductases), and selenium homeostasis (e.g. selenoprotein P) (reviewed in ref. 11 and 12). The embryonic lethal phenotype of mice in which particular components of the selenocysteine-synthetic and decoding machinery were deleted demonstrates the importance of the selenoproteome to the mammalian organism^13^,^14^. Hence, studying how point mutations in *SEPSECS* exert cerebellar dysfunction will shed light onto the role of selenoproteins in the maintenance and development of the human brain.

Comparative analysis of protein sequences showed that these pathological mutations altered conserved residues in SepSecS. While Ala239 and Thr325 are conserved to a reasonable degree, both Tyr334 and Tyr429 are highly conserved in archaea and eukaryotes^3^,^4^. In addition, the crystal structure of human SepSecS^15^ provided a platform for studies on structural and functional effects of these point mutations. Previous structural work has shown that SepSecS is an obligate tetramer held by interactions between two homodimers^15^,^16^,^17^. Although the homodimer interface harbors four seemingly equivalent catalytic pockets and tRNA-binding sites, the enzyme can simultaneously bind, and presumably act on, only two tRNA substrates^15^,^18^. When bound to two tRNAs, one of the SepSecS homodimers always serves as a non-catalytic unit that binds Sep-tRNA^Sec^ and positions the Sep group into the catalytic site of the neighboring catalytic dimer. It has been proposed that the A239T variant of SepSecS most likely lost the ability to bind tRNA^Sec^, whereas the catalytic activity of the Y334C variant is likely diminished^3^,^11^. Furthermore, the premature stop codon in the Tyr429* variant would result in deletion of elements important for both the integrity of the active site and productive binding of tRNA^Sec ^4,11. By contrast, the T325S variant most probably would display only slightly reduced catalytic activity when compared to the wild type (WT) enzyme^4^. However, because predictions derived from structural modeling are limited in their scope, the exact effect(s) of the pathogenic mutations on SepSecS function remained poorly understood. Given that *SEPSECS* mutations are the main cause of these severe cerebellar disorders^3^,^4^, insights into the structure of the mutant SepSecS enzymes may facilitate our understanding of disease etiology and progression.

Herein, we pursued a detailed characterization of four pathogenic variants of SepSecS by X-ray crystallography, transmission electron microscopy, small angle X-ray scattering (SAXS), and other biophysical methods. Our findings show that Y429* expresses at low levels and is completely insoluble, while the A239T, T325S, and Y334C variants are prone to misfolding. The T325S and Y334C variants are capable of forming tetramers that bind tRNA^Sec^, but these tetramers exhibit decreased stability. Our findings suggest that decreased protein stability and the tendency of the SepSecS variants to misfold are the underlying cause of cerebellar atrophy in afflicted patients.

## Results

### Mapping pathogenic mutations onto the structure of human SepSecS

**Figure 1:**
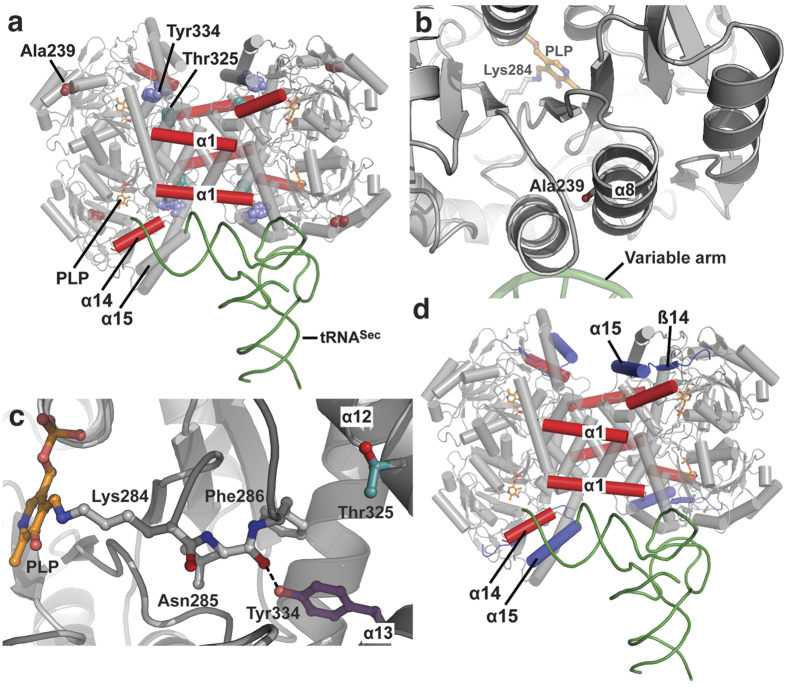
Mapping pathogenic mutations onto the tetrameric human SepSecS-tRNA^Sec^ complex. (**a**) Three missense mutations in SepSecS that cause cerebellar atrophy are shown as spheres: Ala239 is dark red, Thr325 is teal, and Tyr334 is purple. None of the mutations are directly involved in either catalysis or tRNA binding. (**b**) Ala239 (dark red) is in helix α8, near the surface where variable arm of tRNA^Sec^ (green) binds to SepSecS (gray). (**c**) Thr325 (teal) and Tyr334 (purple) are in helices α12 and α13, respectively. Thr325 is ~15Å away from the active site and PLP (orange). The side-chain hydroxyl of Tyr334 forms a H-bond with the backbone carbonyl oxygen of Asn285, and this interaction may stabilize the PLP pocket. (**d**) The non-sense mutation Tyr429* yields truncated protein that lacks elements important for both binding to tRNA^Sec^ and the structure of the active site (purple). tRNA^Sec^ is green, SepSecS is gray and PLP is in gold sticks in all panels.

The crystal structure of human SepSecS allows precise mapping of the mutations implicated in the development of early-onset neurological disorders (Fig. 1a). Genome-wide sequencing identified patients as harboring either compound heterozygous A239T/Y334C or T325S/Y429* or homozygous Y334C mutations in *SEPSECS.* Ala239 is located in helix α8 near the site that interacts with the variable arm of tRNA^Sec^, and is distant from the active site (Fig. 1b). Ala239 is infrequently replaced with Ser in some species and is mostly conserved throughout eukaryotes and archaea^3^. We have previously postulated that the A239T variant would bind tRNA^Sec^ with less affinity compared to WT SepSecS, but that its catalytic function would be unaffected^11^. On the other hand, Thr325 is located in helix α12 and ~15Å away from the active site (Fig. 1c). While conserved in eukaryotes, archaeal orthologs utilize Ser at this position^4^. We hypothesized that the insertion of Ser in position 325 could introduce a kink in α12, thereby altering both the orientation of PLP and the overall conformation of the active site in human SepSecS^4^. These changes would presumably yield a mutant enzyme that binds the tRNA substrate with high affinity, but that is incapable of catalyzing the chemical reaction with the same efficiency as the WT enzyme. The side chain of Tyr334 is in helix α13 near the active-site pocket. This particular residue is highly conserved in eukaryotes and archaea^3^. Its hydroxyl group forms a hydrogen bond with the backbone carbonyl of Asn285, and this interaction may help stabilize a loop that carries Lys284 and the covalently attached PLP cofactor (Fig. 1c). We speculated that the Tyr → Cys mutation would alter the architecture of the active site in the Y334C mutant enzyme, diminishing its catalytic activity but not its ability to bind to tRNA^Sec ^11. Lastly, Tyr429 is located before strand β14. Premature abortion of protein synthesis would yield a truncated enzyme devoid of strand β14, loop β14-α15, and the C-terminal helix α15 (Fig. 1d). Loop β14-α15 establishes a side of the catalytic groove, and helix α15 provides residues that bind the 5′-end of tRNA^Sec ^15. Given these important functions, we hypothesized that the Y429* variant, even if it were to somehow adopt a proper quaternary structure, would not be capable of promoting selenocysteine synthesis^4^.

### Pathogenic variants are less soluble than WT SepSecS

**Figure 2:**
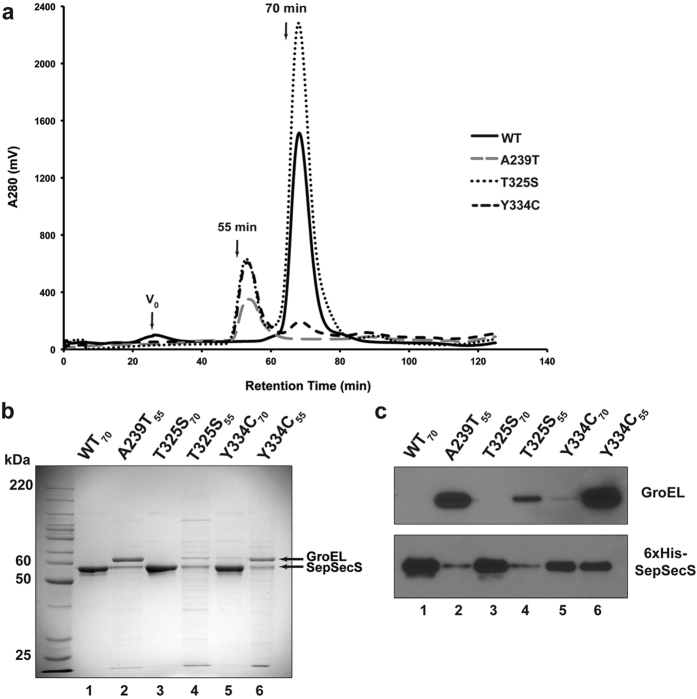
SepSecS A239T, T325S, and Y334C variants co-purify with the bacterial chaperone GroEL. (**a**) Soluble fractions of the WT and pathogenic variants of SepSecS were analyzed by S200 SEC. WT SepSecS elutes as a tetramer in a symmetrical peak at ~70 min (−). The majority of Y334C (−−) elutes ~55 min, with a small fraction forming a tetramer. The entire sample of A239T (−•**−**) elutes at ~55 min, only a small fraction of T325S (•••) forms larger oligomeric species. Only traces recorded at 280 nm are shown for clarity. (**b**) The SDS-PAGE analysis shows that fractions eluting at ~70 min (lanes 1, 3, and 5) primarily contain SepSecS (MW ~60 kDa). The peaks eluting at ~55 min (lanes 2, 4, and 6) are impure and contain predominant bands at ~60 and 65 kDa. (**c**) Western-blot of SEC peaks reveals that the 60-kDa band is the His-tagged SepSecS, and that the 65-kDa band is the bacterial chaperone GroEL. The 70-min peak is SepSecS and the 55-min peak contains both GroEL and SepSecS.

Although the exact location of the mutations was suggestive, their exact effects on the structure and function of the enzyme remained unclear^3^,^4^,^10^. This ambiguity prompted us to pursue biophysical characterization of the SepSecS variants. However, poor expression and insolubility hindered our efforts to obtain pure recombinant mutant protein constructs. Because pathogenic SepSecS variants were insoluble when expressed in BL21(DE3), they were subsequently transformed into SoluBL21(DE3), a bacterial strain engineered to increase solubility of recombinant proteins, and different growth and induction conditions were tested (See Supplementary Table S1). The expression of soluble A239T and Y334C was achieved at +20 °C in LB supplemented with 0.4 M sucrose, whereas Y429* expressed at low levels and as insoluble protein regardless of the incubation temperature, induction point, or the growth media used (Fig. 2). Due to insolubility of Y429*, we focused our efforts on characterizing A239T, T325S, and Y334C mutant enzymes.

### Pathogenic variants of SepSecS co-purify with the bacterial chaperone GroEL

We have previously shown that the functional unit of SepSecS, able to bind tRNA^Sec^ and promote catalysis, is a homotetramer^15^. As pathogenic mutations may alter the structure of this functional core, we set out to determine if the variants can form a stable tetramer in solution.

First, we analyzed the composition of soluble fractions of the SepSecS variants on a size-exclusion chromatography (SEC) column. Our results show that all three variants exhibit an additional peak that elutes significantly earlier (~55 min) than the tetramer peak (~70 min) (Fig. 2a). The A239T variant elutes exclusively in the ‘early’ peak, as does the majority of Y334C. However, a miniscule fraction of Y334C does form tetramers. By contrast, only a small fraction of the T325S mutant enzyme is found in the ‘early’ peak (Fig. 2A.), and this fraction is less than that of A239T and Y334C (Fig. 2b,c).

Subsequently, we analyzed the protein content of both peaks by SDS-PAGE and Western blotting (Fig. 2b,c). The SDS-polyacrylamide (PA) gel revealed that the 70 min peak primarily consists of pure SepSecS, as indicated by a single band at ~60 kDa. However, the ‘early’ peaks of A239T (A239T_55_), Y334C (Y334C_55_), and T325S (T325S_55_) are impure and consist of two major bands migrating at ~60 kDa and ~65 kDa. This led us to speculate that A239T_55_, Y334C_55_, and T325S_55_ contain SepSecS (MW ~60 kDa) that co-purifies with the bacterial chaperonin, GroEL (MW ~65 kDa). To test this, we performed Western blot analyses using anti-GroEL and anti-His antibodies (Fig. 2c). The blots confirmed that the ~60 kDa band is indeed SepSecS, that the ~65 kDa band is GroEL, and that GroEL is present in the ‘early’ peaks only. In contrast, the same analysis of the tetramer fractions of the WT, T325S_70_ and Y334C_70_ did not reveal the presence of GroEL.

**Figure 3:**
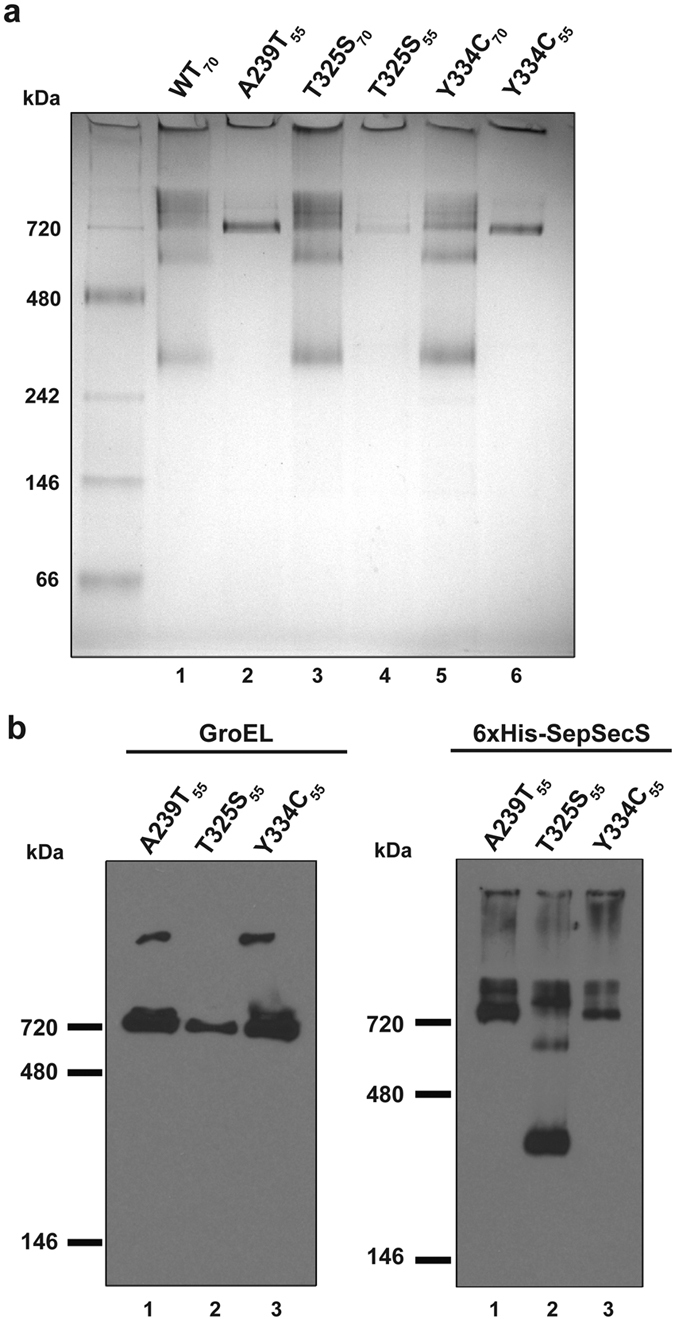
Native PAGE and Western blots indicate that A239T and Y334C variants form a stable complex with GroEL. (**a**) Native PAGE analysis of S200 SEC elution fractions reveals that fractions of WT, T325S, and Y334C that elute at ~70 min (lanes 1, 3, and 5) consist of SepSecS tetramers (band between 242–480 kDa), a ~600-kDa oligomer, and other species of higher molecular weight. Fractions eluting at ~55 min (lanes 2, 4, and 6) form a larger species of molecular weight of ~720 kDa. (**b**) Fractions of A239T, T325S and Y334C eluting at ~55 min were resolved on the native gel and then probed with anti-GroEL (left panel) and anti-His antibodies (right panel). The 720-kDa band consists of the bacterial GroEL and SepSecS suggesting that misfolded pathogenic variants are bound GroEL. The ‘early’ peak of T325S contains a smaller fraction of GroEL in addition to tetrameric SepSecS. Notably, in both the native gel and blots a substantial portion of protein does not enter the gel, suggesting that there may be higher order oligomers or aggregates species.

Further, we analyzed both the 55 min and 70 min peaks by blue-native (BN) PAGE. The aim was to visualize the oligomerization status of the variants and whether they are bound to GroEL. The WT SepSecS and T325S_70_ and Y334C_70_ assemble into tetramers, indicated by the band between 242–480 kDa (theoretical tetramer MW ~230 kDa). These fractions also exhibit higher-order oligomers (MW >480 kDa), which appear as a ladder of bands extending to the top of the gel. In contrast, the A239T_55_, T325S_55_, and Y334C_55_ samples contain a single band at ~720 kDa (Fig. 3a). Western blotting using anti-GroEL antibodies confirmed that this ~720 kDa band contains GroEL (Fig. 3B). Probing for His-tagged SepSecS in these fractions revealed that all variants exhibit a ladder-like appearance near the top of the gel, similar to the pure tetrameric samples (compare right panels in Fig. 3b and Supplementary Fig. S1). However, in A239T_55_ and Y334C_55_ a distinct band at ~720 kDa that coincides with GroEL is present, thus implying that A239T and Y334C are bound to GroEL. Because A239T and Y334C do not exhibit bands between 242–480 kDa, we speculated that these variants are misfolded and thus incapable of assembling into functional tetramers. On the other hand, the banding pattern for T325S_55_ almost exactly matches that of the WT SepSecS, T325S_70_, and Y334C_70_ (Fig. 3b, lane 2), suggesting that T325S_55_ adopts a tetrameric structure over time. It is also important to mention that both the native gels and Western blots indicated the presence of protein in the wells of the gel, implying that SepSecS variants may form even larger species (e.g. aggregates, oligomers) that are not amenable to electrophoretic analyses (Fig. 3 and Supplementary Fig. 1). Lastly, Western blots of the native, but not SDS-PA gels (see above), showed that WT, T325S_70_ and Y334C_70_ contain some GroEL as well. However, unlike the SepSecS_55_ fractions, the tetramers do not stably associate with GroEL (Supplementary Fig. S1). Hence, we conclude that small quantities of GroEL represent the impurity that co-purified with SepSecS tetramers.

**Figure 4:**
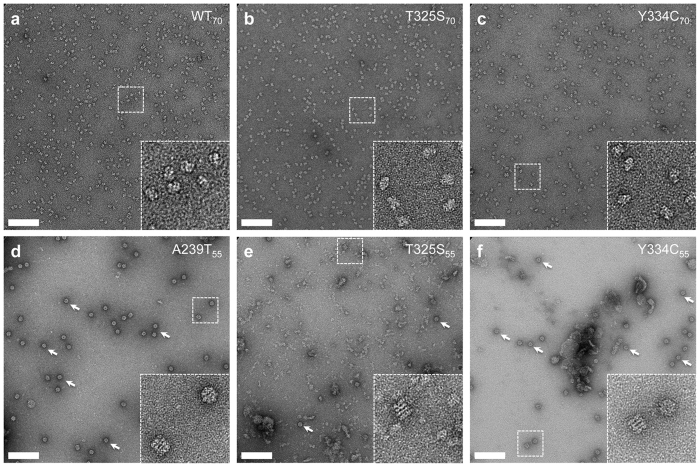
Negative-stain electron microscopy of SepSecS WT, A239T, T325S and Y334C. (**a–c**) Representative images of 70 min peak fractions of WT (**a**), T325S (**b**), and Y334C (**c**) indicating homogenous SepSecS tetramers. (**d–f**) Representative image of 55 min peak fractions of A239T (**d**), T325S (**e**), and Y334C (**f**) showing various degrees of GroEL particles as indicated by arrows. Insets are shown on the bottom right of each image and scale bars are 100 nm.

The visualization of the content of the 55 min and 70 min peaks using negative stain electron microscopy further corroborated our conclusions (Fig. 4). Namely, the 70 min peaks of WT, T325S, and Y334C contained homogenous SepSecS tetramers (Fig. 4a–c), whereas the 55 min peaks of A239T, T325S, and Y334C showed particles whose size and shape are consistent with GroEL (Fig. 4d–f) and in relative amounts consistent with that observed on the SDS-PA gel (Fig. 2b). The A239T_55_ and Y334C_55_ samples almost exclusively showed these GroEL particles. The T325S_55_ sample was more heterogeneous with aggregates and vesicular like particles with only a few GroEL particles.

### Thr325Ser and Tyr334Cys do not affect the binding affinity of the SepSecS-tRNA complex

We have determined that, on average, one or two tRNAs bind to one SepSecS tetramer at a time^18^. In this study we asked whether tetramers of T325S and Y334C are capable of binding tRNA^Sec^, in a similar manner as the WT enzyme. It is important to note that because A239T did not form stable tetramers, further biophysical characterization of this particular variant was not pursued. Because SepSecS binds unacylated tRNA^Sec^ equally well as Sep-tRNA^Sec ^19, and since it is challenging to synthesize stable Sep-tRNA^Sec^ in large quantities only unacylated tRNA^Sec^ was used in this study.

**Figure 5:**
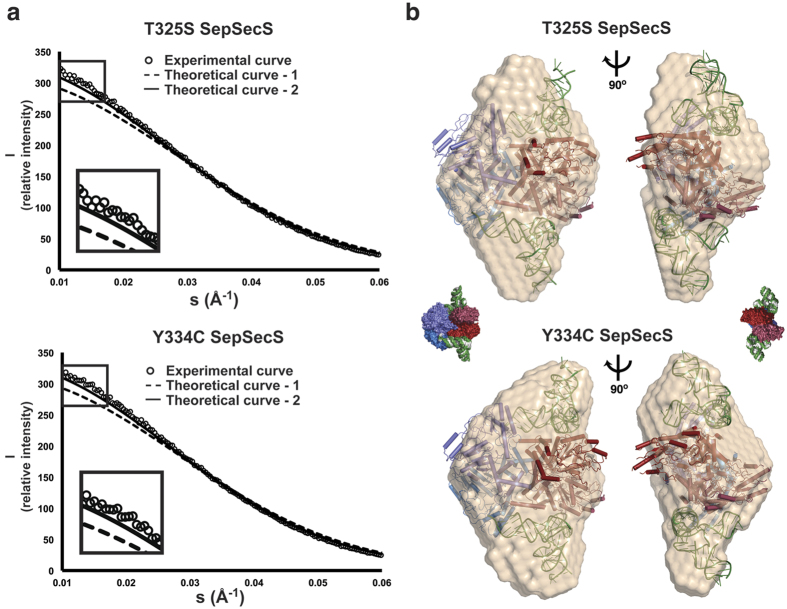
Characterization of the T325S SepSecS:tRNA^Sec^ and Y334C SepSecS:tRNA^Sec^ binary complexes by SAXS. (**a**) The overlay of SAXS curves suggests that T325S (top) and Y334C (bottom) bind two tRNA^Sec^ molecules in solution. Theoretical curves calculated for the WT SepSecS in complex with one (−−) and two (−) tRNA^Sec^ molecules are shown. The experimental curves (○) were obtained for T325S:tRNA^Sec^ and Y334C:tRNA^Sec^ using 6:1 molar excess of tRNA^Sec^ over SepSecS tetramers. (**b**) The superimpositioning analysis reveals an excellent agreement between the crystal structure of the WT SepSecS-tRNA^Sec^ complex^15^ (red, blue, and green ribbon and surface diagrams) and the experimental SAXS envelopes (beige) of T325S:tRNA^Sec^ (top) and Y334C:tRNA^Sec^ (bottom). Two views related by 90° rotation around the vertical axis are shown; surface representation diagrams of the binary complex serve to orient the reader.

To determine the stoichiometry of the mutant binary complexes we coupled a size-exclusion chromatography column to a SAXS detector (SEC-SAXS)^18^. The Guinier plot of the SAXS scattering data revealed the absence of higher-order oligomers and aggregates in both the T325S-tRNA^Sec^ and Y334C-tRNA^Sec^ samples (Fig. 5). Our data show that T325S-tRNA^Sec^ and Y334C-tRNA^Sec^ complexes have a radius of gyration (R_g_) of 47.92 ± 1.56 Å and 47.31 ± 1.72 Å, respectively. These values are in agreement with the experimental R_g_ of 48.54 Å for the WT binary complex^18^. To distinguish between the two possibilities, we compared theoretical and experimental scattering curves for complexes containing various molar ratios between SepSecS and tRNA^Sec^. Our results demonstrate that the experimental scattering curve derived from the mutant complexes superimposes well onto the theoretical scattering curve for SepSecS in complex with two tRNAs (Fig. 5a). Moreover, the crystal structure of the WT complex fits well into the SAXS envelopes of the mutant complexes (Fig. 5b). This argues that the previously observed arrangement of tRNAs bound to SepSecS and the architecture of the complex^15^,^18^ is preserved in the T325S and Y334C variant tetramers. Based on the SAXS data, the T325S and Y334C mutations do not significantly alter the stoichiometry and architecture of the resulting binary complexes with tRNA^Sec^.

### Tetramers of T325S and Y334C adopt the same structure as WT SepSecS

To establish whether mutations T325S and Y334C alter the structure of SepSecS, we solved crystal structures of holo T325S and the T325S-tRNA^Sec^ and Y334C-tRNA^Sec^ complexes (See Supplementary Table S2). The binary complex crystals belonged to the trigonal space group and contained two SepSecS dimers and one tRNA^Sec^ molecule in the asymmetric unit, whereas those of the holo T325S were of the orthorhombic space group with half of the SepSecS tetramer in the asymmetric unit (See Supplementary Table S2).

A global structural comparison showed that T325S and Y334C tetramers adopt the same structure as the WT enzyme (See Supplementary Fig. S2). When all Cα atoms of the WT SepSecS were superimposed onto T325S and Y334C, low r.m.s.d. values of 0.97 Å and 0.98 Å were obtained, respectively. Inspection of the initial Fo-Fc electron density difference maps confirmed that Thr325 was substituted with Ser in the T325S variant (See Supplementary Fig. S3) and that Tyr334 was replaced with Cys in Y334C (See Supplementary Fig. S2). A closer inspection of the crystal structure revealed that the Thr325 → Ser replacement does not cause any changes in the tetrameric structure of SepSecS (See Supplementary Fig. S1). Furthermore, we expected that replacing Tyr334 with a much shorter Cys residue would remove a hydrogen bond between the side chain of Tyr334 and the backbone carbonyl oxygen of Asn285 (See Supplementary Fig. S2). This, in turn, could have resulted in repositioning of the PLP cofactor and presumably reduced catalytic activity of SepSecS. Surprisingly, in the Y334C crystal, the side chain of Cys334 coordinates two water molecules, which interact with the backbone carbonyl of Asn285 in the same fashion as the Tyr side chain in the WT enzyme (See Fig. S2). Based on these observations, we conclude that pathogenic mutations Thr325Ser and Tyr334Cys do not alter the three-dimensional structure of the SepSecS tetramer.

### Pathogenic variants, T325S and Y334C, are less stable than the WT SepSecS

Our results suggest that T325S and Y334C mutations do not significantly alter the structure of the mutant tetramers or the stoichiometry and architecture of the binary complexes. The question then remains as to what caused the loss of SepSecS activity in patients harboring these variants. We hypothesized that T325S and Y334C could affect the overall stability of the variant tetramers.

**Figure 6:**
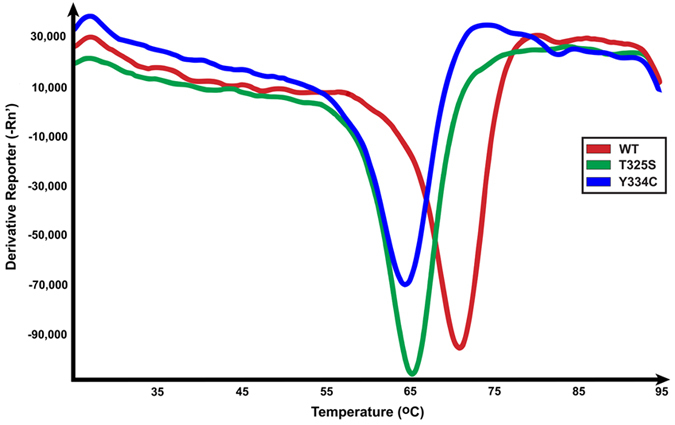
Pathogenic variants of SepSecS are less stable than the WT enzyme. Melting curves for tetrameric WT SepSecS (red), T325S (green), and Y334C (blue) measured by differential scanning fluorimetry are shown. Curves have similar shapes, but the T_m_ of T325S and Y334C is ~5 °C lower than that of WT SepSecS.

To test this possibility, we used differential scanning fluorimetry (DSF) to evaluate the melting curves of the tetrameric fractions of WT_70_, T325S_70_, and Y334C_70_ SepSecS (Fig. 6, Table 1). The shape of the resulting melting curves for the variants is almost indistinguishable from that of the WT enzyme, but the corresponding melting temperatures (T_m_) differ significantly (Fig. 6). The WT enzyme has a T_m_ value of 70.6 ± 1.0 °C. While the tetrameric T325S_70_ and Y334C_70_ have T_m_ values that are lower by ~5 °C with T325S_70_ having a T_m_ value of 65.0 ± 0.8 °C and Y334C_70_ having T_m_ of 64.6 °C ± 0.0 °C (Table 1). We conclude that the Thr325Ser and Tyr334Cys mutations decrease the stability of SepSecS tetramers. Hence, the increased propensity of mutant enzymes to misfold and the decreased stability of the mutant tetramers may decrease the fraction of SepSecS molecules that can support selenoprotein synthesis.

## Discussion

Recent studies identified mutations in human *SEPSECS* as the cause of the development of severe disorders characterized by cerebellar atrophy^3^,^4^. Patients harboring A239T and Y334C mutant alleles and patients carrying T325S and Y429* all exhibited severe, progressive, cerebellar atrophy and other neurological damage^3^,^10^. Both studies suggested that loss of SepSecS activity may be the underlying cause of the observed pathological conditions, but there is little understanding for the mechanism of disease progression. In this study we examined how the pathogenic mutations in human *SEPSECS* affect the structure of the enzyme.

Our results revealed that the pathogenic SepSecS variants–A239T, T325S, and Y334C–are significantly less soluble than the WT enzyme. With the exception of Y429*, which never expressed to measurable quantities, expression of soluble variants required use of a specialized bacterial strain. Further, our results demonstrate that the majority of soluble A239T and Y334C associate with the bacterial chaperonin, GroEL, which is necessary for the proper folding of many bacterial proteins. Additionally, in a recombinant protein expression system GroEL may bind to the nascent recombinant protein to facilitate proper folding and to prevent accumulation of potentially toxic aggregates of misfolded proteins in the cell. Co-purification with GroEL suggests that the pathogenic variants of SepSecS have an increased propensity to misfold. The degree to which each of the variants co-purifies with GroEL was different, and thus could be used as an indirect measure of protein folding capability. For instance, A239T cannot be purified in the absence of GroEL, implying that this variant cannot fold into its native structure under the conditions tested. Indeed, soluble A239T does not adopt a tetrameric structure, a hallmark of catalytic SepSecS. In contrast, because only a small fraction of the T325S variant co-purifies with GroEL, it is likely the least damaging of the pathogenic variants. In contrast to the A239T variant, T325S contains a significant proportion of tetramers, and its fraction that co-elutes with GroEL is capable of adopting a tetrameric structure over time. While T325S in many ways resembles WT SepSecS, it is important to stress that this behavior was observed only when utilizing a bacterial strain engineered to enhance recombinant protein solubility (i.e. SoluBL21). Interestingly, tetramers of T325S and Y334C adopt the same structure. Taken together our data suggest that the A239T, T325S, and Y334C are likely to be completely inactive, partially active, and marginally active *in vivo*, respectively. This analysis is in good agreement with the results of an indirect activity assay in the *E. coli* system that is based on the substitution of the bacterial selenocysteine synthase with the mutant human SepSecS enzyme. This activity assay indicated the A239T and Y334C mutations completely ablated enzyme activity, while the T325S mutation modestly diminished SepSecS activity^3^,^4^. However, since tRNA^Sec^ is essential for mammalian development, it is reasonable to suggest that Y334C most likely exhibits marginal activity *in vivo* to explain the viability of the homozygous Y334C patients^13^. Furthermore, patients compound heterozygous for the T325S/Y429* exhibited a 15–40% decrease in selenoprotein levels compared to healthy patients. These data suggest that disease-causing mutations partially, but not completely, abolished SepSecS activity. In the case of the nonsense mutation, Y429*, we showed that SepSecS-Y429* expresses at extremely low levels as insoluble protein under all conditions tested. It is likely that the Y429* mRNA is targeted for the nonsense-mediated mRNA decay, while any truncated nascent polypeptide is targeted to the proteasome for degradation. A previous report using the *E. coli* activity assay found the Y429* variant to be completely inactive^4^.

Our studies enabled us to understand how particular mutations may affect the structure of SepSecS and selenoprotein synthesis. A239T and Y429* variants of SepSecS are most likely completely inactive due to their insolubility and inability to form productive tetramers. In contrast, T325S and Y334C are thought to be partially active since they are capable of forming soluble SepSecS tetramers that mimic the WT structure. The autosomal recessive inheritance of *SEPSECS* mutations in patients and the relative health of heterozygous parents harboring a single mutant allele suggest that one copy of WT SepSecS is sufficient for the development of a healthy organism. While loss of SepSecS activity would appear to be the most obvious effect of the mutations that contributes to disease pathology, this may not be their only detrimental effect. Native blot analysis of the GroEL-bound A239T and Y334C SepSecS variants suggested that they were misfolded and devoid of any tetrameric SepSecS. Thus, misfolding-induced cellular toxicity by the SepSecS variants may also play a role in disease pathology, especially considering that the brain is particularly susceptible to protein misfolding disorders^20^,^21^. This is reminiscent of our recent report in which we suggest that misfolding of human glutaminyl-tRNA synthetase (GlnRS) may be the cause of the early-onset neurodegeneration^22^. Although unrelated enzymes, SepSecS and GlnRS are vital for protein synthesis with an important distinction is that GlnRS plays a more general role since glutamine is found in almost all proteins, whereas selenocysteine is limited to only 25 members of the proteome. Nevertheless, our results suggest that misfolding of enzymes involved in tRNA aminoacylation and protein synthesis could significantly impact both the development and maintenance of the brain.

Many questions still remain as to how decreased SepSecS stability contributes to disease progression. The importance of selenium to the brain is evident from animal studies demonstrating that the brain sequesters selenium at the expense of other organs during times of selenium deficiency^23^,^24^. This suggests that, although not dependent on a high level of selenium, the brain does require maintenance of selenium homeostasis at all times^25^. Still it is unclear why the cerebellum appears more susceptible to SepSecS mutations compared to other brain regions. A recent study, utilizing a neuronal-specific knockout of the murine tRNA^Sec^ gene, yielded a phenotype characterized by severe cerebellar atrophy^26^. A more specific knockout of the *GPX4* gene resulted in a less severe, cerebellar phenotype, suggesting that cerebellar development and maintenance may require particular selenoproteins. However, these studies did not directly examine the role of SepSecS in brain development. Additional studies investigating the status of the selenoproteome in cellular and mouse models in which *SEPSECS* is mutated are necessary.

Lastly, general questions about how defects in RNA processing contribute to PCH pathology remain unanswered. To date, there are 10 types of PCH. Mutations in 8 gene products, including SepSecS, which are involved in RNA processing and aminoacylation of tRNA have been implicated as the cause of PCH. Mutations in the *EXOSC3* gene, which encodes for a subunit of the RNA exosome, are responsible for PCH1B^1^. Multiple subtypes of PCH2 and PCH4 are caused by mutations in *TSEN* genes that encode components of the tRNA splicing endonuclease complex^2^. PCH6 is caused by mutations in *RARS2*^5^,^6^,^7^, which encodes the enzyme that charges mitochondrial arginine tRNA. Finally, PCH10 is caused by mutations in *CLP1*, a component of both the tRNA splicing endonuclease complex and the pre-mRNA cleavage complex II^8^,^9^. Given that differential diagnosis of PCH is challenging due to the early onset of the disorder and rather uncharacteristic clinical presentation of symptoms, this list will almost certainly expand in the future. Indeed, at the time of writing additional two reports of autosomal-recessive cerebellar disorders due to mutations in *SEPSECS*, one of which detailed late-onset (adolescence) cerebellar degeneration, have been published^27^,^28^. Currently, there are 12 known *SEPSECS* mutations that have contributed to disorders of cerebellar atrophy (See Supplementary Table S3) causing much ambiguity in the literature regarding the nomenclature of *SEPSECS* phenotypes. A more unified understanding of the neurological phenotype caused by autosomal recessive inheritance of *SEPSECS* mutations would greatly aid understanding of disease progression and aid diagnosis.

Our study facilitates understanding of these childhood neurological disorders by demonstrating that disease-causing *SEPSECS* mutations destabilize protein structure and increase the propensity of SepSecS to misfold. Misfolding likely diminishes the catalytic activity of SepSecS and selenoprotein levels. Both insufficient selenoprotein levels and misfolded SepSecS may be responsible for the observed atrophy of cerebellar and cerebral structures. Further cellular and animal studies are necessary to delineate the roles of selenoprotein synthesis and misfolding in disease progression and understand why the cerebellum is particularly susceptible to mutations in *SEPSECS.*

## Methods

### Cloning, expression and purification of SepSecS variants

Human *SEPSECS* was cloned into the pQE80 vector with an N-terminal His-tag. SepSecS variants, A239T, Y334C, T325S, and Y429* were generated using a QuikChange Site-Directed Mutagenesis kit (Agilent Technologies) (See Supplementary Table S4) following the manufacturer’s protocol, and mutations were confirmed by Sanger sequencing. Constructs were transformed into BL21 and SoluBL21 (Genlantis) *E. coli* expression strains. Cultures were grown in SoluBL21 Minimal medium, LB, LB +0.4 M sucrose, Terrific broth, Super broth, and 2xYT medium in the presence of ampicillin. Protein expression was induced with 0.5 or 1 mM IPTG (isopropyl-beta-D-thiogalactopyranoside) at different cell densities, and cultures were incubated at +15 °C, +20 °C or +37 °C (Table S1). To determine the level of protein solubility, cells were lysed by B-PER (Thermo Scientific) or sonication, and the soluble and insoluble fractions were separated by centrifugation and analyzed on a denaturing gel. SepSecS variants were purified from the soluble fraction and stored as previously described^15^.

### *In vitro* transcription and purification of tRNA^Sec^

The gene encoding human tRNA^Sec^ was cloned, amplified by PCR, and *in vitro* transcribed as described^15^. Briefly, the transcription reaction was performed at +37 °C for 3 h in the buffer containing 40 mM Tris, pH 8.1, 22 mM MgCl_2_, 10 mM DTT, 1mM spermidine, 0.01% Triton X-100, 50 μg/mL BSA, 10 mM GMP, 2 mM ribonucleotides, the PCR product of the tRNA^Sec^ gene (20 ng/μl), and 160 μM T7 RNA polymerase. The cleared reaction was applied to a Resource-Q column (GE-Healthcare), and tRNA^Sec^ was purified using a linear gradient of NaCl (0.2–1.0 M) in 20 mM Tris, pH 8.1. Following elution, the human tRNA^Sec^ was purified on a S200 16/60 Superdex size-exclusion column (SEC) (GE-Healthcare) equilibrated with 20mM Tris, pH 8.1, and 150mM NaCl. The purified tRNA^Sec^ was flash frozen in liquid nitrogen and stored at −80 °C.

### SDS- and Native-PAGE analysis

The expression level and purity of WT and SepSecS variants were evaluated by using standard 8% discontinuous SDS-PA gel electrophoresis. The elution peaks from the SEC that contained SepSecS variants were analyzed using 6% discontinuous, BN Tris-glycine PA gels. To aid entry of proteins into the gel, Coomassie G-250 was added to the cathode buffer to a final concentration of 0.002%. Protein bands were visualized by Coomassie Brilliant Blue R-250 staining.

### Western blot analysis

SepSecS mutant enzymes were resolved either on SDS- or native PA gels and transferred onto Low Fluorescence 0.2 μm PVDF membrane (GE Healthcare). For SDS-PAGE blots either 25 or 10 ng of total protein was loaded onto the gel for probing for either the 6xHis-tag or GroEL, respectively. For native PAGE blots, either 3 μg or 350 ng of total protein was loaded for probing for the 6xHis-tag or GroEL, respectively. The membrane was blocked in blocking buffer (5% nonfat dry milk in PBST, 50 mM phosphate buffer, pH 7.6, 150 mM NaCl and 0.1% Tween-20) for 1 h at room temperature. Subsequently, the membrane was incubated with either an anti-His-tag antibody conjugated with horseradish peroxidase (R&D Systems) or anti-GroEL (Sigma-Aldrich) in blocking buffer overnight at 4 °C. Following primary antibody incubation, the GroEL blot was incubated with an HRP-conjugated goat anti-rabbit antibody (Sigma-Aldrich). Immunoblots were developed by enhanced chemiluminescence using ECL Prime Western Detection Reagent (GE Healthcare) and a Konica Minolta SRX-101A imager with Amersham Hyperfilm ECL (GE Healthcare).

### Electron Microscopy

SepSecS samples were first diluted to ~0.04 mg/mL and ~0.4 mg/mL for the 70 and 55 min fractions from the size exclusion chromatography column, respectively. They were then adsorbed to a freshly glow-discharged carbon-film grid for 15s, and stained with 0.7% uranyl formate. Images were collected on an FEI Tecnai T12 equipped with a 4k × 4k Gatan Ultrascan CCD camera at an image pixel size of ~0.18 nm.

### Small angle X-ray scattering (SAXS)

Samples containing tRNA^Sec^ and either T325S or Y334C (with the molar ratio of six tRNA^Sec^ molecules per SepSecS tetramer) were mixed to yield a final concentration of ~3.5 mg/mL in 20mM Tris, pH 8.0, 15 0mM NaCl, 5% (v/v) glycerol, 0.5 mM TCEP, and 10 μM PLP. SAXS experiments were conducted at the 18-ID Biophysics Collaborative Access Team beam-line (BioCAT), Advanced Photon Source, Argonne National Laboratory (APS-ANL), Chicago, IL^29^. Samples were exposed to X-rays using an in-line setup in which a 24 ml S200 column was directly coupled to the SAXS cell. The scattering data were collected every 2 s with 1.0 s exposure. On average, 600–800 data points were measured covering the time before, during, and after elution of each peak. Measurements taken before and after peak elution were used to establish the baseline scattering. IGOR Pro (WaveMetrics, Inc., Lake Oswego, OR) and ATSAS package^30^ were used for data reduction and processing, respectively. The Guinier Analysis and calculation of the radius of gyration, R_g_, were done in PRIMUS^31^,^32^. Radius of gyration and the pair-distance distribution function, P(r), were calculated from the entire scattering pattern using GNOM^31^,^33^, the low-resolution *ab initio* models were calculated in DAMMIF^34^, and model clustering and averaging was done in DAMCLUST^35^. SUPCOMB was used to superimpose the SAXS *ab initio* models onto the X-ray crystal structure^35^. Finally, theoretical SAXS curves derived from the crystal structure were generated and overlaid with the experimental data in CRYSOL^36^.

### Crystallization and structure determination

Crystals of holo T325S, T325S-tRNA^Sec^, and Y334C-tRNA^Sec^ were obtained by the sitting drop vapor-diffusion method at +12 °C. The crystals of holo T325S were grown in 0.28–0.34 M lithium citrate and 16–18% (w/v) PEG 3,350, while those of the T325S-tRNA^Sec^ binary complex were obtained from 0.1 M sodium cacodylate trihydrate (Hampton Research), pH 6.4, 0.24 M lithium citrate, and 10–12% (w/v) PEG 3,350. The Y334C-tRNA^Sec^ complex crystallized in 0.1 M sodium cacodylate trihydrate, pH 6.0, 0.24 M lithium citrate, and 8% (w/v) PEG 3,350. Crystals were cryoprotected with 20% ethylene glycol prior to X-ray exposure. Data sets were collected at liquid nitrogen temperature at the Life Sciences Collaborative Access Team (LS-CAT) beamline at APS-ANL. The X-ray diffraction data were processed in HKL-2000^37^. Data sets from three isomorphous crystals of the holo SepSecS-T325S were merged and the crystal structure was determined by molecular replacement in Phaser^38^ using the crystal structure of the WT SepSecS (PDB ID 3HL2) as a search model. Similarly, the WT SepSecS-tRNA^Sec^ complex was used for the initial refinement of the T325S SepSecS-tRNA^Sec^ and Y334C SepSecS-tRNA^Sec^ complexes. Structure refinement was performed in Phenix^39^, and model building was done in Coot^40^,^41^. All figures were produced in PyMOL^42^.

### Differential scanning fluorimetry

SepSecS samples (1–20 μM) were mixed with Sypro Orange (Sigma; 1:500 dilution) in a 1:4 ratio in 10 μL of buffer containing 20 mM Tris, pH 8.0, 150 mM NaCl, 5% (v/v) glycerol, 0.5 mM TCEP, and 10 μM PLP. Mixtures were dispensed in triplicates into a 384-well microplate and analyzed on the Vii^TM^ 7 Real-Time PCR System (Life Technologies) using the Melting Curve method with continuous heating (0.075 °C/s) from +25 °C to +95 °C. Melting curves were recorded in real time as the change in fluorescence signal and analyzed using the ViiA^TM^ 7 RUO Software (Life Technologies).

## Additional Information

**Accession Codes:** The coordinates and structure factors are deposited in PDB with the accession codes 4ZDL (for holo T325S SepSecS), 4ZDO (for T325S SepSecS:tRNA^Sec^) and 4ZDP (for Y334C SepSecS:tRNA^Sec^).

**How to cite this article**: Puppala, A. K. *et al*. Structural basis for early-onset neurological disorders caused by mutations in human selenocysteine synthase. *Sci. Rep.* **6**, 32563; doi: 10.1038/srep32563 (2016).

## Supplementary Material

Supplementary Information

## Figures and Tables

**Table 1:** Melting temperature (T_m_) for the tetrameric SepSecS enzymes.

Enzyme	T_m_1 (°C)	T_m_2 (°C)	T_m_3 (°C)	Average T_m_ (°C)
WT SepSecS	69.55	71.20	71.20	70.65 ± 0.95
T325S	65.40	65.60	64.10	65.00 ± 0.81
Y334C	64.60	64.60	64.60	64.60 ± 0.00
