# Supplementary material for: Structural basis for early-onset neurological disorders caused by mutations in human selenocysteine synthase
Source: Sci Rep. 2016 Aug 31;6:32563. doi: 10.1038/srep32563 (PMC5006159; doi:10.1038/srep32563)
Supplement: Supplementary Information [file srep32563-s1.pdf]

SUPPLEMENTARY INFORMATION

Structural basis for early-onset neurological disorders caused by mutations in human selenocysteine synthase

Anupama K. Puppala[1], Rachel L. French[1,#], Doreen Matthies[2], Ulrich Baxa[2], Sriram Subramaniam[2], Miljan Simonović[1]*

Author affiliation:

[1] Department of Biochemistry and Molecular Genetics, University of Illinois at Chicago, Chicago, Illinois 60607

[2] Laboratory of Cell Biology, Center for Cancer Research, National Cancer Institute, National Institutes of Health, Bethesda, MD 20892

* To whom correspondence should be addressed:

Miljan Simonović
Associate Professor
Department of Biochemistry and Molecular Genetics
University of Illinois at Chicago
E-mail: msimon5@uic.edu

900 S. Ashland Ave., 1354 MBRB
Chicago, IL 60607
U.S.A.
Phone: (312) 996-0059
Fax: (312) 413-0353

# Current address:

Department of Biochemistry and Molecular Biology, St. Louis University, St. Louis, MO 63104

SUPPLEMENTARY TABLES:

Table S1. Conditions used for test expressions of Y334C, A239T and Y429* mutants of human SepSecS in SoluBL21.

Growth temperature	[IPTG]	OD_{600} level at induction	Expression temperature	Additional notes
37°C<br>37°C	0.1mM<br>0.5mM	1.0<br>0.5	15°C<br>15°C	
37°C	0.5mM	0.5	15°C	Heat shock culture at +42°C for 20 minutes prior to addition of IPTG.
37°C<br>37°C	0.1mM<br>0.5mM	1.0<br>0.5	20°C<br>20°C	
37°C	0.5mM	0.5	20°C	Heat shock culture at +42°C for 20 minutes prior to addition of IPTG.
37°C<br>37°C	0.1mM<br>0.5mM	1.0<br>0.5	37°C<br>37°C	
37°C	0.5mM	0.5	37°C	Heat shock culture at +42°C for 20 minutes prior to addition of IPTG.

Table S2. Data collection and refinement statistics.

	Holo T325S	T325S-tRNA^{Sec}	Y334C-tRNA^{Sec}
**Data Collection**			
Space group	$P2_12_12$	$P3_112$	$P3_112$
Unit cell dimensions: a, b, c (Å) α, β, γ (°)	68, 83, 193	166, 166, 238 γ=120	167, 167, 240 γ=120
Resolution range (Å)	50.00 – 2.26 (2.4-2.26)	50.00 – 2.65 (2.80-2.65)	42.00 – 2.70 (2.80-2.7)
Unique reflections*	44,237 (2,201)	133,557 (6,063)	98,427 (9,248)
R_{sym} or R_{merge} (%)	19.0	15.8	11.8
I/ σ(I) *	12.8 (1.1)	13.4 (1.1)	11.8 (2.0)
Completeness (%)*	95.0 (77.0)	99.8 (97.1)	93.8 (88.7)
Redundancy*	7.6 (2.7)	9.0 (5.3)	3.2 (3.0)
**Refinement**			
Resolution range (Å)	50.00 – 2.49	50.00 – 2.65	42.00 – 2.70
Average B-factor:	44.6	59.8	42.8
Macromolecule	44.1	60.1	43.0
Ligands	40.4	41.4	36.9
Solvent	50.5	57.1	35.5
Number of residues:			
Protein	890	1,899	1,851
Nucleic acid	N/A	87	87
Non-hydrogen atoms:	7,564	18,619	17,458
Macromolecule	6,881	17,340	16,963
Ligands	30	60	70
Water	646	1,219	425
R_{work}*	16.3 (26.4)	23.2 (37.9)	19.8 (28.1)
R_{free}*	22.1 (31.0)	27.2 (43.0)	22.9 (32.7)
RMS deviations:			
Bonds (Å)	0.008	0.014	0.014
Angles (°)	1.11	1.01	1.16
Ramachandran plot:			
Favored (%)	95.4	94.4	96.4
Allowed (%)	4.6	5.0	3.6

* Statistics for the highest-resolution shell are shown in parentheses.

Table S3. Summary of known *SEPSECS* mutations.

Genetic Mutation	Observed Inheritance	Mutation	Amino Acid Change	Phenotype	Year reported
c.1001A>G<br>c.715G>A	Compound heterozygous	Missense	p.Tyr334Cys<br>p.Ala239Thr	PCH2D	2010 [1]
c.1001A>G	Homozygous	Missense	p.Tyr334Cys	PCH2D	2010 [1]
c.1466A>T	Homozygous	Missense	p.Asp489Val	PCH2D	2014 [2]
c.1027_1120 del.	ND	Frameshift	p.Glu343Leufs*2	Neurodegeneration	2015 [3]
c.1A>G<br>c.388+3A>G	Compound heterozygous	Missense/ splicing (frameshift)	p.Met1Val?	PCH2D	2015 [4]
c.974C>G<br>c.1287C>A	Compound heterozygous	Missense	p.Thr325Ser<br>p.Tyr429*	Progressive encephalopathy	2015 [5]
c.1001T>C	Homozygous	Missense	p.Tyr334His	PCH2D	2016 [6]
c.77delG<br>c.356A>G	Compound heterozygous	Frameshift/ missense	p.Arg26Profs*42<br>p.Asn119Ser	Late-onset PCCA	2016 [7]
c.356A>G<br>c.467G>A	Compound heterozygous	Missense	p.Asn119Ser<br>p.Arg156Gln	Late-onset PCCA	2016 [8]

Table S4. Site-directed mutagenesis primer sequence information.

Variant[#]	Primer[&]	Primer Sequence[$]
A239T	FWD	GCCTGATAGATTAGAAGAACTGACTGTGATTTGTGCTAATTATGA
	REV	TCATAATTAGCACAAATCACAGTCAGTTCTTCTAATCTATCAGGC
T325S	FWD	CACCTTCTTTAGATGTCCTTATTTCTT
	REV	ATTTGATCCAAGTGACAATAAAGAAAT
Y334C	FWD	ATTGTCACTTGGATCAAATGGCTGTAAGAAGCTACTAAAAGAAAGAA
	REV	TTCTTTCTTTTAGTAGCTTCTTACAGCCATTTGATCCAAGTGACAAT
Y429*	FWD	AGAGGCTTTATGTCACATACAAATAAT
	REV	GAGGTAAGCACAAGGCTAATTATTTG

[#] - All variants were generated using the WT SepSecS gene cloned in the pQE-80 vector.

[&] - FWD, forward; REV, reverse;

[$] - All primer nucleotide sequences are listed in the 5’ to 3’ direction.

SUPPLEMENTARY FIGURES:

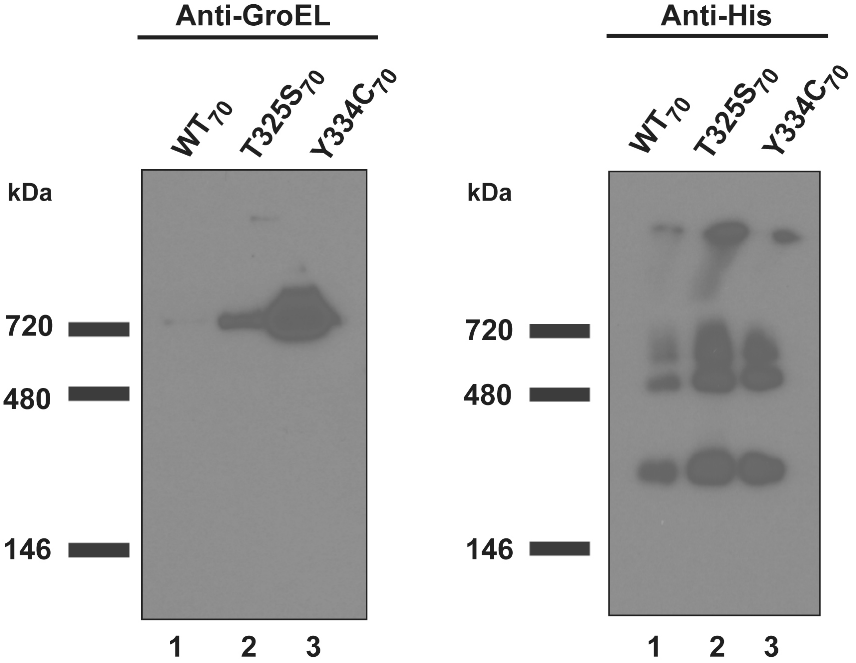

Figure S1. Analysis of tetramers of WT, T325S and Y334C that elute at 70 min by Native PAGE and Western blots. Fractions of WT (lane 1), $T325S_{70}$ (lane 2) and $Y334C_{70}$ (lane 3) eluting at ~70 min were resolved on the native gel and then probed with anti-GroEL (left panel) and anti-His (right panel) antibodies. The SepSecS tetramers contain GroEL (band at 720 kDa) that does not associate with tetramers. Also, all tetrameric fractions are capable of forming ordered oligomeric structures as evidenced by bands between 480 and 720 kDa in lanes 1-3 in the right panel.

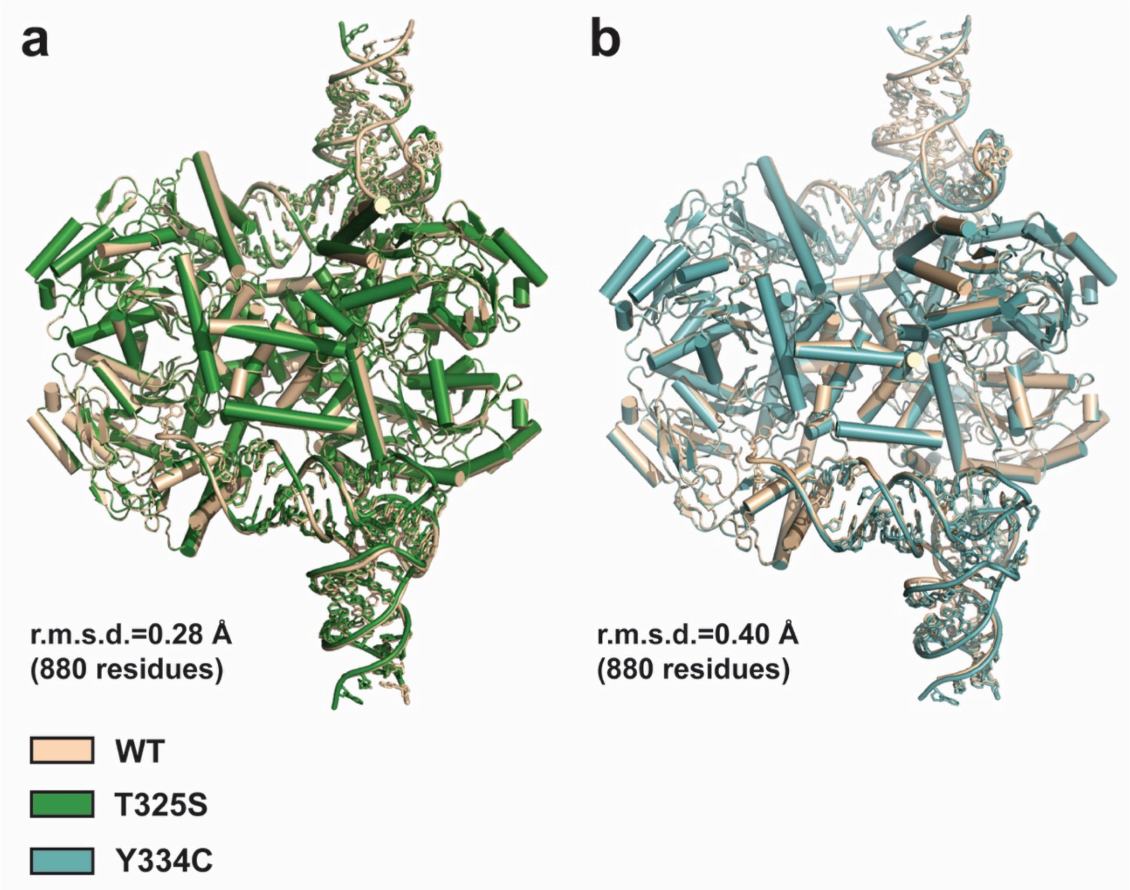

Figure S2. The binary complexes of T325S and Y334C with tRNA^{Sec} are similar to the structure of the WT complex*.* A structural comparison of T325S:tRNA^{Sec} (**a**) and Y334C:tRNA^{Sec} (**b**) to the WT complex reveals that pathogenic mutants adopt the same structure and bind tRNA^{Sec} in the same manner as the WT enzyme. The superimpositioning of structures yielded low r.m.s.d. values of 0.28 (T325S:tRNA^{Sec}) and 0.40 Å (Y334C:tRNA^{Sec}). T325S:tRNA^{Sec}, Y334C:tRNA^{Sec} and the WT complex are colored green, light blue and beige, respectively.

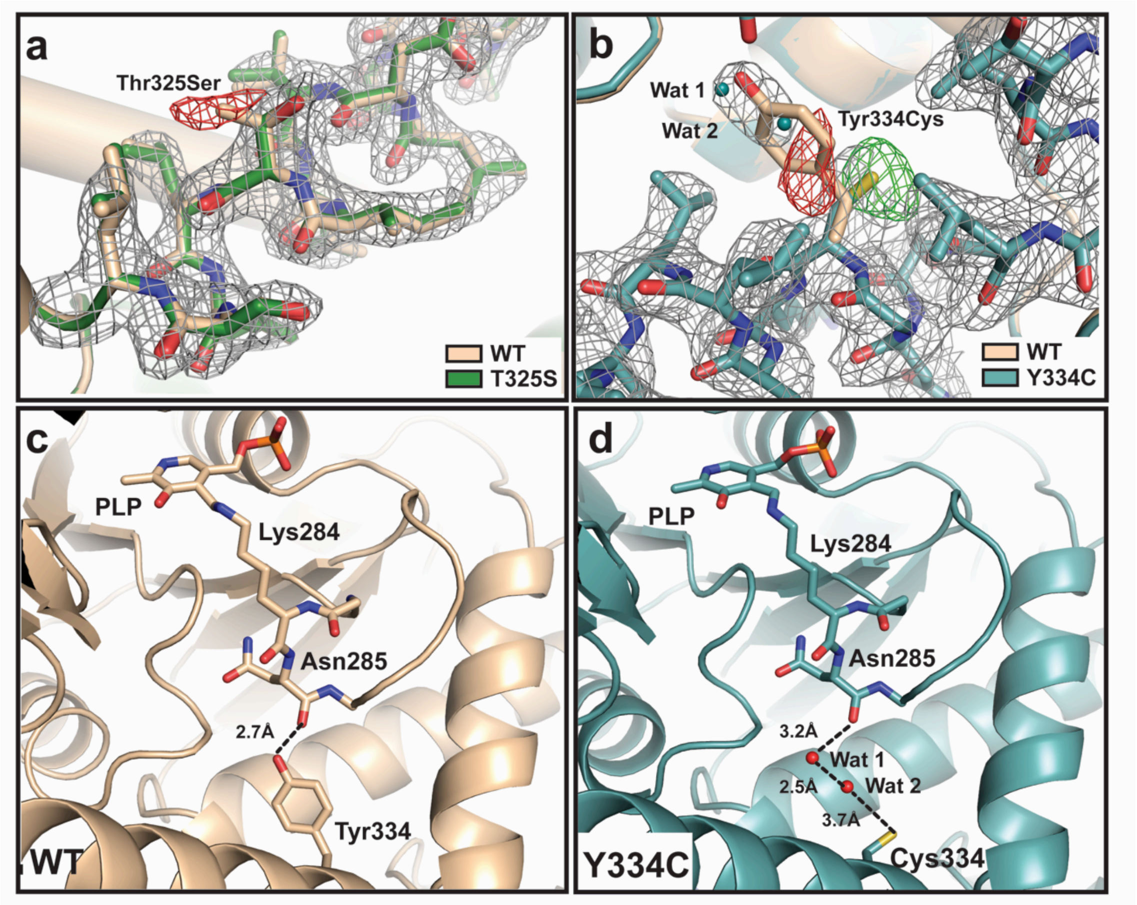

Figure S3. T325S and Y334C mutations do not perturb the structure of SepSecS. (**a**) The excerpt of helix α12, which harbors the Thr325Ser mutation, shows that the WT (beige) and T325S (green) structures bound to $tRNA^{Sec}$ are nearly identical. The final 2mFo-DFc electron density map (grey mesh) is countered at 1.5σ. The negative peak in the mFo-DFc map (red mesh; 3.0σ) confirms that Thr325 is replaced with Ser**.** Both maps were calculated to 2.65-Å resolution. (**b**) Close up view of helix α13 and the active site shows that the Y334C tetramer (light blue) adopts the same structure as the WT enzyme (beige). The 2mFo-DFc map (gray mesh) agrees well with the final model. The positive (green mesh) and negative (red mesh) peaks of the mFo-DFc map confirm that the Tyr334 side chain is replaced with Cys and two water molecules (light blue spheres). Maps were calculated to 2.7 Å and contoured at 4.0 (2mFo-DFc) and 2.7σ (mFo-DFc). (**c**) In the WT SepSecS (beige), Tyr334 stabilizes the loop harboring the PLP cofactor through a H-bond with the backbone carbonyl of Asn285. (**d**) In Y334C (light blue), the thiol group of Cys (yellow) and two water molecules (red spheres) establish a network of H-bonds with the backbone carbonyl oxygen of Asn285, which anchors Lys284 and PLP.

SUPPLEMENTARY REFERENCES:

1 Agamy, O. *et al.* Mutations disrupting selenocysteine formation cause progressive cerebello-cerebral atrophy. *Am J Hum Genet* **87**, 538-544 (2010).

2 Makrythanasis, P. *et al.* Diagnostic exome sequencing to elucidate the genetic basis of likely recessive disorders in consanguineous families. *Hum Mutat* **35**, 1203-1210 (2014).

3 Alazami, A. M. *et al.* Accelerating novel candidate gene discovery in neurogenetic disorders via whole-exome sequencing of prescreened multiplex consanguineous families. *Cell Rep* **10**, 148-161 (2015).

4 Eggens, V. R. *et al.* EXOSC3 mutations in pontocerebellar hypoplasia type 1: novel mutations and genotype-phenotype correlations. *Orphanet J Rare Dis* **9**, 23, doi:10.1186/1750-1172-9-23 (2014).

5 Anttonen, A. K. *et al.* Selenoprotein biosynthesis defect causes progressive encephalopathy with elevated lactate. *Neurology* **85**, 306-315 (2015).

6 Pavlidou, E. *et al.* Pontocerebellar hypoplasia type 2D and optic nerve atrophy further expand the spectrum associated with selenoprotein biosynthesis deficiency. *Eur J Paediatr Neurol* **20**, 483-488 (2016).

7 Iwama, K. *et al.* Milder progressive cerebellar atrophy caused by biallelic SEPSECS mutations. *J Hum Genet*, doi:10.1038/jhg.2016.9 (2016).

8 Chiba, T., Iwama, A. & Yokosuka, O. Cancer stem cells in hepatocellular carcinoma: Therapeutic implications based on stem cell biology. *Hepatol Res* **46**, 50-57 (2016).
